# T-cell activation or tolerization: the Yin and Yang of bacterial superantigens

**DOI:** 10.3389/fmicb.2015.01153

**Published:** 2015-10-20

**Authors:** Aline Sähr, Sandra Förmer, Dagmar Hildebrand, Klaus Heeg

**Affiliations:** Medical Microbiology and Hygiene, Department of Infectious Diseases, University Hospital HeidelbergHeidelberg, Germany

**Keywords:** superantigen, anergy, Treg, class II signaling, co-inhibitory molecules, STAT3, IDO, PD-L1

## Abstract

Bacterial superantigens (SAg) are exotoxins from pathogens which interact with innate and adaptive immune cells. The paradox that SAgs cause activation and inactivation/anergy of T-cells was soon recognized. The structural and molecular events following SAg binding to antigen presenting cells (APCs) followed by crosslinking of T-cell receptors were characterized in detail. Activation, cytokine burst and T-cell anergy have been described *in vitro* and *in vivo*. Later it became clear that SAg-induced T-cell anergy is in part caused by SAg-dependent activation of T-regulatory cells (Tregs). Although the main focus of analyses was laid on T-cells, it was also shown that SAg binding to MHC class II molecules on APCs induces a signal, which leads to activation and secretion of pro-inflammatory cytokines. Accordingly APCs are mandatory for T-cell activation. So far it is not known, whether APCs play a role during SAg-triggered activation of Tregs. We therefore tested whether in SAg (Streptococcal pyrogenic exotoxin A) -treated APCs an anti-inflammatory program is triggered in addition. We show here that not only the anti-inflammatory cytokine IL-10 and the co-inhibitory surface molecule PD-L1 (CD274) but also inhibitory effector systems like indoleamine 2,3-dioxygenase (IDO) or intracellular negative feedback loops (suppressor of cytokine signaling molecules, SOCS) are induced by SAgs. Moreover, cyclosporine A completely prevented induction of this program. We therefore propose that APCs triggered by SAgs play a key role in T-cell activation as well as inactivation and induction of Treg cells.

## Introduction

Superantigens (SAg) are bacterial exotoxins which share unique immunological properties. SAg released by staphylococci or streptococci during infection or even colonization induce a strong activation of the immune system. Major hallmark of this activation is the fulminant release of cytokines (Carlsson and Sjögren, 1985) leading to a disastrous cytokine storm (Miethke et al., 1992; Michie et al., 1994) which might lead to an uncontrolled systemic shock with high lethality. The toxic shock syndrome (TSS) was recognized to be mediated by a SAg, the TSS toxin 1, TSST-1 (Miethke et al., 1993). In the meantime, many bacterial exotoxins have been classified as SAgs (Fraser and Proft, 2008), including the erythrogenic toxins of *Streptococcus pyogenes* (SPEA, SPEC) and the enterotoxins from *Staphlococcus aureus* (Lina et al., 2004).

Superantigen behave like bifunctional agents: the bind to conserved regions of MHC class II molecules and to V-beta encoded regions of the T-cell receptor (TCR) (Choi et al., 1990; Dellabona et al., 1990). Crosslinking class II on APC with TCR induces T-cell activation with subsequent cytokine release. While all SAg bind to class II, the binding to the TCR is V-beta specific, thus single SAg activates a V-beta defined subfraction of the T-cell pool (Kappler et al., 1989). Nevertheless, in the human system a single SAg can activate 1 to 10% of the T-cell pool, leading to a strong oligoclonal T-cell response which exceeds the clone size activated by a protein antigen at least by the factor of 1000 (Herrmann and MacDonald, 1991).

The tracking of SAg reactive T-cells using the V-beta TCR expression has greatly facilitated the analyses of SAg-induced T-cell responses. After initial activation accompanied with secretion of cytokines including TNF, interleukin-2 (IL-2) and IFN-gamma a phase of clonal T-cell expansion follows which is then succeeded by apoptosis and clonal retraction (Herrmann et al., 1992; Lee and Vitetta, 1992; Huang and Crispe, 1993; Miethke et al., 1995). The apoptosis is not completely, roughly 50% of the initial numbers of V-beta bearing T-cells survive. However, these T-cells fail to respond to further stimulation, i.e., display an anergic phenotype (MacDonald et al., 1993; Wahl et al., 1993). Unfolding of unresponsiveness is prevented in the presence of the T-cell immunosupressive agent cyclosporine A (CsA) but not rapamycin (Vanier and Prud’homme, 1992; Prud’homme et al., 1995). This was taken as an indication that anergy induction is dependent on calcineurin and is triggered primarily in T-cells. Besides anergy induction it was also shown that after stimulation with SAg the fraction of CD4^++^CD25^+^ foxp3^+^ Treg within the T-cell pool is significantly augmented (Wang et al., 1998; Papiernik, 2001; Feunou et al., 2003; Grundström et al., 2003; Ivars, 2007). The cellular interactions and signaling pathways leading to Treg induction after SAg stimulation are not well understood.

For T-cell activation, the presence of APC and the binding of SAg to their MHC class II molecules are mandatory (MacDonald et al., 1993; Rink et al., 1997). The binding regions of SAg to MHC class II have been studied and characterized in detail. It became evident that SAg not only binds to class II but also share the ability at different levels to crosslink MHC class II molecules (Hudson et al., 1995; Kozono et al., 1995). This suggested that SAg might confer a signal to the MHC class II expressing APC. Indeed MHC class II signaling after crosslinking has been observed in B-cells and stem cells which was characterized by activation of tyrosine kinases like Syk (Mooney et al., 1990, 1994; Scholl and Geha, 1994; Kanner et al., 1995; Yamaguchi et al., 1999). In monocytes binding of SAg and thus crosslinking of MHC class II molecules was followed by an intracellular increase of Ca^2+^ which was then succeeded by activation and eventually secretion of pro-inflammatory cytokines like TNF (Palkama and Hurme, 1993; Trede et al., 1993a,b; Mehindate et al., 1995; Espel et al., 1996; Khan et al., 2008). Interestingly Treg seem to utilize class II signaling too. Mature Tregs express LAG-3 (CD223) which has been shown to bind to MHC class II and to crosslink subsequently the molecules (Hemon et al., 2011).

Since SAg-mediated stimulation of T-cells includes activation as well as tolerization processes we hypothesized that both events are determined by respective cellular or humoral events triggered in APC by SAg. We therefore tested whether co-inhibitory molecules (such as PD-L1; Francisco et al., 2009, 2010), negative signaling circuits (such as suppressor of cytokine signaling molecules, SOCS; Alexander, 2002; Strebovsky et al., 2011), or inhibitory effector systems (such as IDO; Lestage et al., 2002; Hill et al., 2007) are induced by SAg in APC.

## Materials and Methods

### Reagents

Streptococcal pyrogenic exotoxin A (SPEA) was purchased from Toxin Technology Inc. (Sarasota, FL, USA). The mTOR–inhibitor rapamycin (50 nM), NFAT–inhibitor VIVIT (1 M), PI3–kinase–inhibitor Wortmannin (50 nM) and piceatannol (50 μM), a Syk–inhibitor were acquired from Calbiochem (Schwalbach, Germany). CsA was purchased from R&D Systems (Wiesbaden, Germany).

### Isolation of Primary Cells

Human peripheral blood mononuclear cells (PBMCs) were isolated from fresh blood by density gradient centrifugation (Pancoll 1.077 g/ml; PAN Biotech). Monocytes were isolated via CD14 MicroBeads (Miltenyi Biotech, Bergisch-Gladbach, Germany) with the autoMACS separator. 2 × 10^6^ cells were cultured in RPMI 1640 (Sigma–Aldrich, Taufkirchen, Germany) supplemented with 100 IU/mL of penicillin, 100 μg/mL streptomycin containing 10% heat inactivated fetal calf serum (Promocell, Heidelberg, Germany) at 37°C in a humidified atmosphere in the presence of 5% CO_2_ and stimulated for 24 h.

### Flow Cytometry

Twenty four hours after stimulation monocytes were analyzed for the surface markers CD14 (clone TÜK4), CD80 (clone L307.4), CD86 (clone IT2.2), PD-L1 (clone MIH1) and MHC-II (clone Tu39). Analyses were performed on a FACS Canto I (BD Biosciences).

The antibodies were purchased from Becton Dickinson (Heidelberg, Germany), except PD-L1 (eBioscience, Frankfurt/Main, Germany).

### Western Blotting

8 × 10^6^ cells were lysed 24 h after stimulation in RIPA lysis buffer containing protease inhibitor cocktail and phosphatase inhibitor cocktail from Roche (Mannheim, Germany). Equal amounts of the lysates were fractionated by SDS-PAGE and electrotransferred to nitrocellulose membranes. After blocking and washing steps the indicated antibodies, purchased from Cell Signaling Technology (Danvers, MA, USA), were incubated for 24 h and detected via chemiluminescence (ECL; Perkin Elmer, Groningen, Netherlands).

### ELISA

Cell-free supernatants were harvested 24 h after stimulation and analyzed for IL–6, IL–10, IL–12p40 and TNF by commercial available ELISA kits from Becton Dickinson (OptEIA; Becton Dickinson, Heidelberg, Germany) according to the manufacturer’s instructions.

#### Statistics

Statistical significance was assessed using SPSS statistics software and paired student’s *t*-test evaluation with ^∗^: *p* ≤ 0.05, ^∗∗^: *p* ≤ 0.005. Further on we confirmed the results with Multiple Comparisons of Means (Tukey Contrasts), performed with R. Significance codes: 0 ‘^∗∗∗^’ 0.001 ‘^∗∗^’ 0.01 ‘^∗^’ 0.05 ‘.’ 0.1 ‘ ’ 1. When the results of the methods differ, significance of Tukey’s test is shown.

### RNA Purification and Quantitative Real – Time PCR

Total RNA from 4 × 10^6^ cells was isolated using the High Pure RNA isolation Kit (Roche, Mannheim, Germany), cDNA was synthesized from equal amounts of RNA using the first strand cDNA kit from Thermo Scientific (Waltham, MA USA). Quantitative real–time RT-PCR was performed using ABsoluteTM QPCR SYBR^®^Green Low ROX Mix (Thermo Scientific, Waltham, MA, USA). Relative expression was calculated by normalization to β–Actin mRNA expression levels as 2-ΔCt. All primers were synthesized by Eurofins MWG Operon (Ebersberg, Germany) (**Table 1**).

**Table 1:** Primers used.

Gene	Forward – primer	Reverse – primer
ß-Actin	aga gct acg agc tgc ctg ac	agc act gtg ttg gcg tac ag
calcineurin	aaa cag tga ctg gcg cat c	ccg gct tac agc aaa aga ag
IDO	tta gag tca aat ccc tca gtc c	ttt gca gat ggt agc tcc tc
IL-1b	agc tga tgg ccc taa aca ga	gca tct tcc tca gct tgt cc
IRF-1	gct ggg aca tca aca agg at	tgg tct ttc acc tcc tcg at
JNK	gca tgg gct aca agg aaa ac	ttc agg aca tgg tgt tcc aa
p38	gac aca aaa acg ggg tta cg	tgg gtc acc aga tac aca tca
p44/42	agt aca tcc act ccg cca ac	cgt agc cac ata ctc cgt ca
CD274	tgc tgt ctt tat att cat gac cta c	tcc tcc att tcc caa tag aca
SOCS1	tcc ccc tca acc ccg t	cat ccg ctc cct cca acc
SOCS3	ggg agt ccc ccc aga aga g	ata gga gtc cag gtg gcc gt
STAT1	ccg ttt tca tga cct cct gt	ggc gtt ttc cag aat ttt cc
STAT3	cag gtt gct ggt caa att cc	tgt gtt tgt gcc cag aat gt
TDO	ggt tcc tca ggc tat cac tac c	cag tgt cgg gga atc agg t

## Results

### Superantigens Induce Cytokine Secretion in APC

**FIGURE 1:**
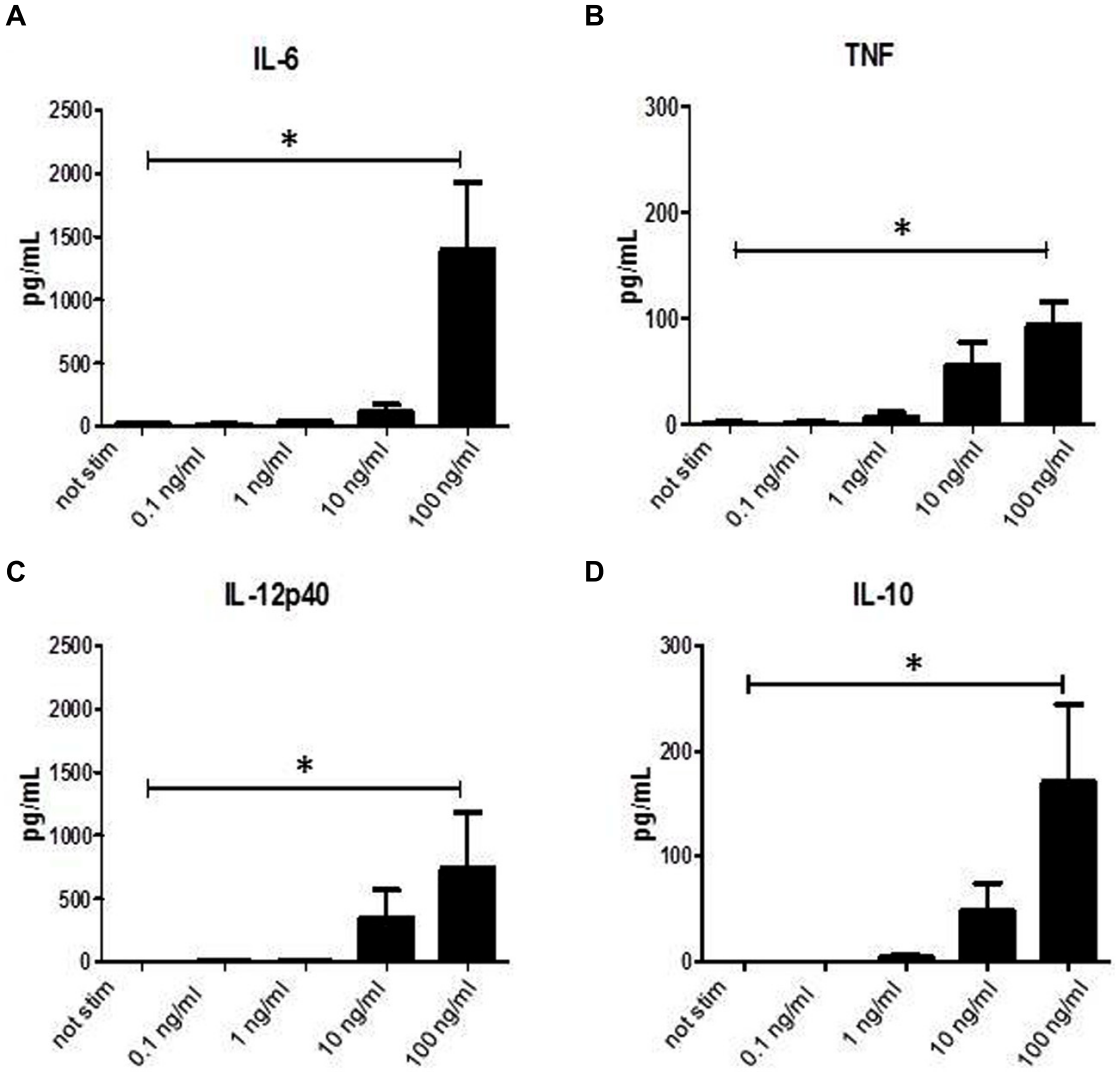
**Streptococcal pyrogenic exotoxin A (SPEA)- induced cytokines**. CD14^+^ monocytes were stimulated with increasing concentrations of SPEA. 24 h after stimulation supernatants were analyzed by ELISA for **(A)** IL-6, **(B)** TNF, **(C)** IL-12p40 and **(D)** IL-10 (mean ± SD, *n* = 5). Statistical analysis was performed using a Multiple Comparison of Means (Tukey Contrasts). Significance code: 0 ‘^∗∗∗^’; 0.001 ‘^∗∗^’; 0.01 ‘^∗^’.

It has been shown previously that binding of SAg to MHC class II molecules induces activation and secretion of cytokines. When we incubated monocytes for 24 h with graded doses of SPEA a dose dependent induction of cytokine secretion was found (**Figures 1A–C**). Not only pro-inflammatory cytokines like IL-6, TNF, or IL-12p40 were induced but also secretion of anti-inflammatory IL-10 could be recorded (**Figure 1D**).

**FIGURE 2:**
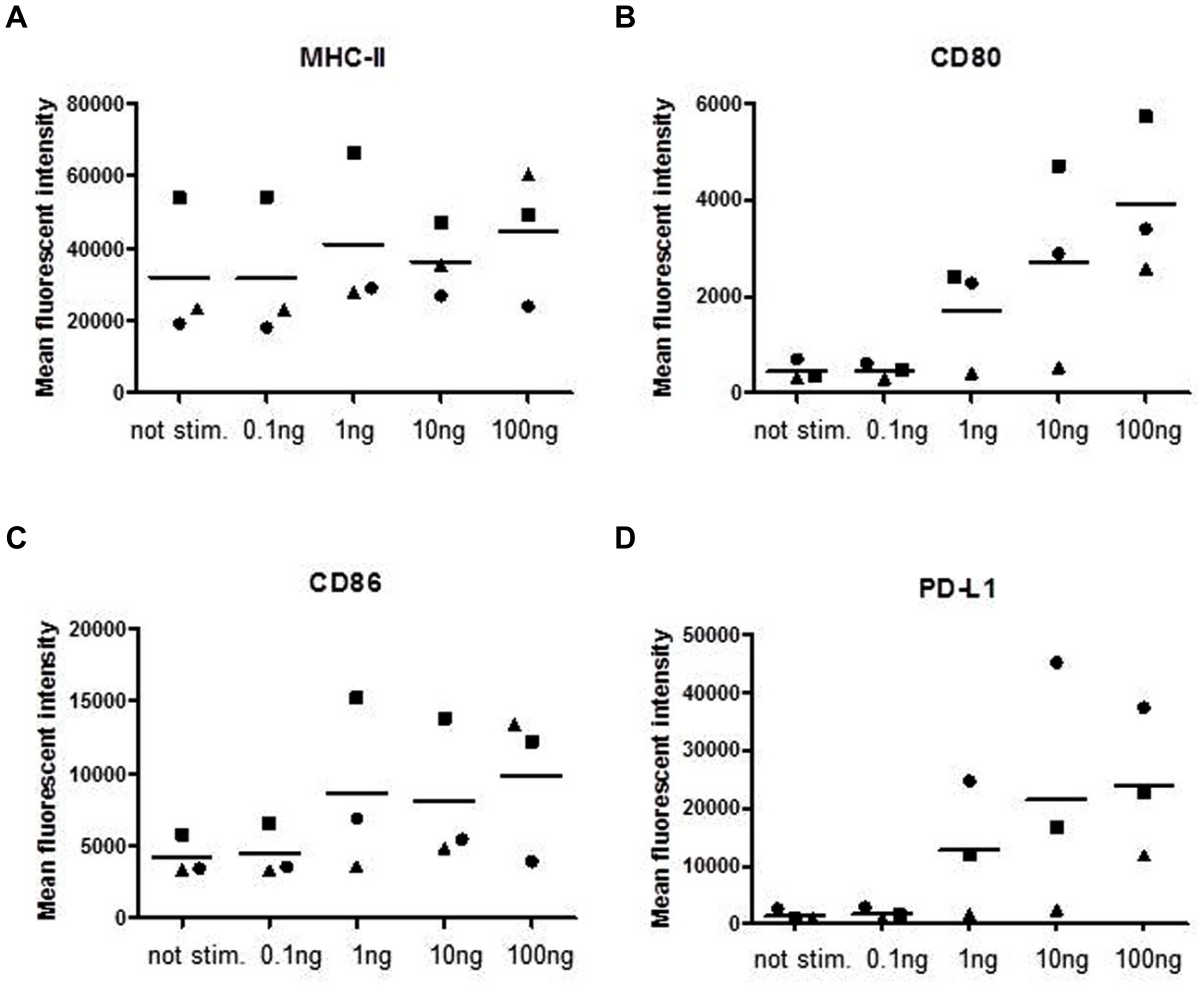
**Streptococcal pyrogenic exotoxin A- induced surface molecules**. CD14^+^ monocytes were stimulated with increasing concentrations of SPEA as indicated. 24 h after stimulation cells were harvested and analyzed for surface markers by flow cytometry **(A)** MHC-II, **(B)** CD80, **(C)** CD86 and **(D)** PD-L1. The mean fluorescent intensity of three different experiments is shown. The line represents the mean of the experiments.

Next we assayed for the expression of surface molecules involved in antigen presentation and T-cell interaction (**Figure 2**). The surface expression of MHC class II molecules showed no dependency on SPEA stimulation (**Figure 2A**), while a dose-dependent expression of the co-stimulatory molecules CD80 and CD86 was observed (**Figures 2B,C**). Interestingly the co-inhibitory molecule PD-L1 (CD274) was strongly induced (**Figure 2D**, Supplementary Figure S1).

### Superantigens Induce mRNA Expression of Inhibitory Pathways

**FIGURE 3:**
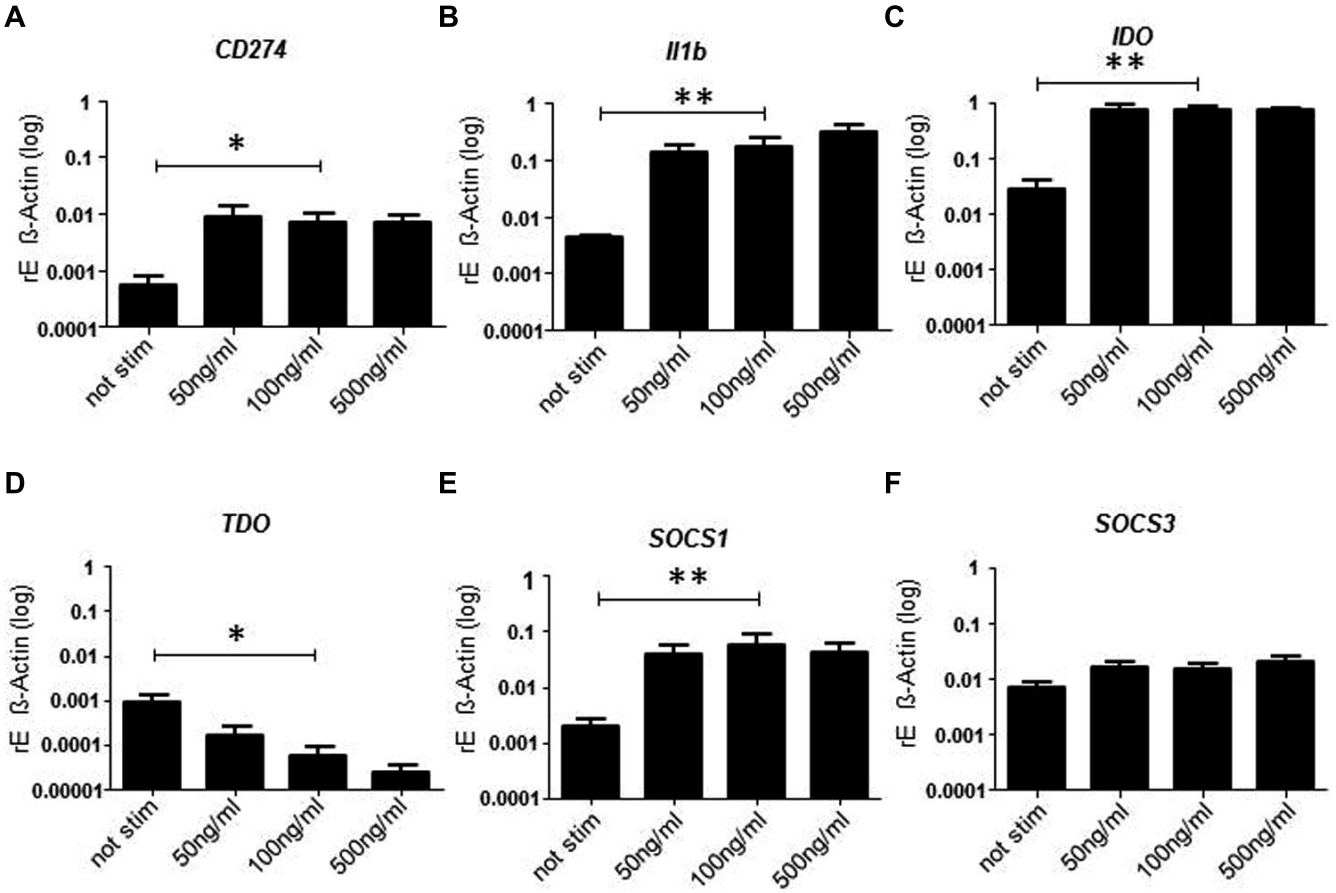
**Streptococcal pyrogenic exotoxin A- induced mRNA expression of inhibitory pathways**. CD14^+^ monocytes were stimulated 24 h with increasing concentrations of SPEA. Quantitative real-time PCR of cDNA was performed for **(A)** CD274, **(B)** Il1b, **(C)** IDO, **(D)** TDO, **(E)** SOCS1, and **(F)** SOCS3. Shown is the mean of induction compared to ß-Actin (±SD) of 5 donors. Statistical analysis was performed using a Multiple Comparison of Means (Tukey Contrasts). Significance code: 0 ‘^∗∗∗^’; 0.001 ‘^∗∗^’; 0.01 ‘^∗^’.

Since PD-L1 expression indicated that inhibitory pathways might be induced we analyzed the mRNA expression of molecules involved in negative regulatory circuits (**Figure 3**). As expected mRNA expression of PD-L1 was significantly enhanced after treatment with SPEA (**Figure 3A**). The same was true for IL-1beta (**Figure 3B**). As IL-1 beta is produced as inactive precursor we additionally confirmed the elevated release of the active cytokine (Supplementary Figure S2). Surprisingly expression of the inhibitory effector enzyme IDO was observed (**Figure 3C**). In contrast, expression of tryptophan 2,3-dioxygenase (TDO) was suppressed (**Figure 3D**) suggesting an inverse regulation. Within APCs negative feedback loops have been identified which regulate the response to cytokine stimulation. Molecules of the SOCS family play a pivotal role during these processes. We therefore analyzed the expression of SOCS1 and SOCS3 after stimulation of monocytes with SAg. While SOCS1 showed a clear induction, SOCS3 was only slightly induced (**Figures 3E,F**). mRNA expression of other signaling molecules was unchanged except for STAT1 and IRF-1which showed a slight increased expression (Supplementary Figure S3).

### Signaling Pathways Involved in SAg-Mediated Activation of APC

**FIGURE 4:**
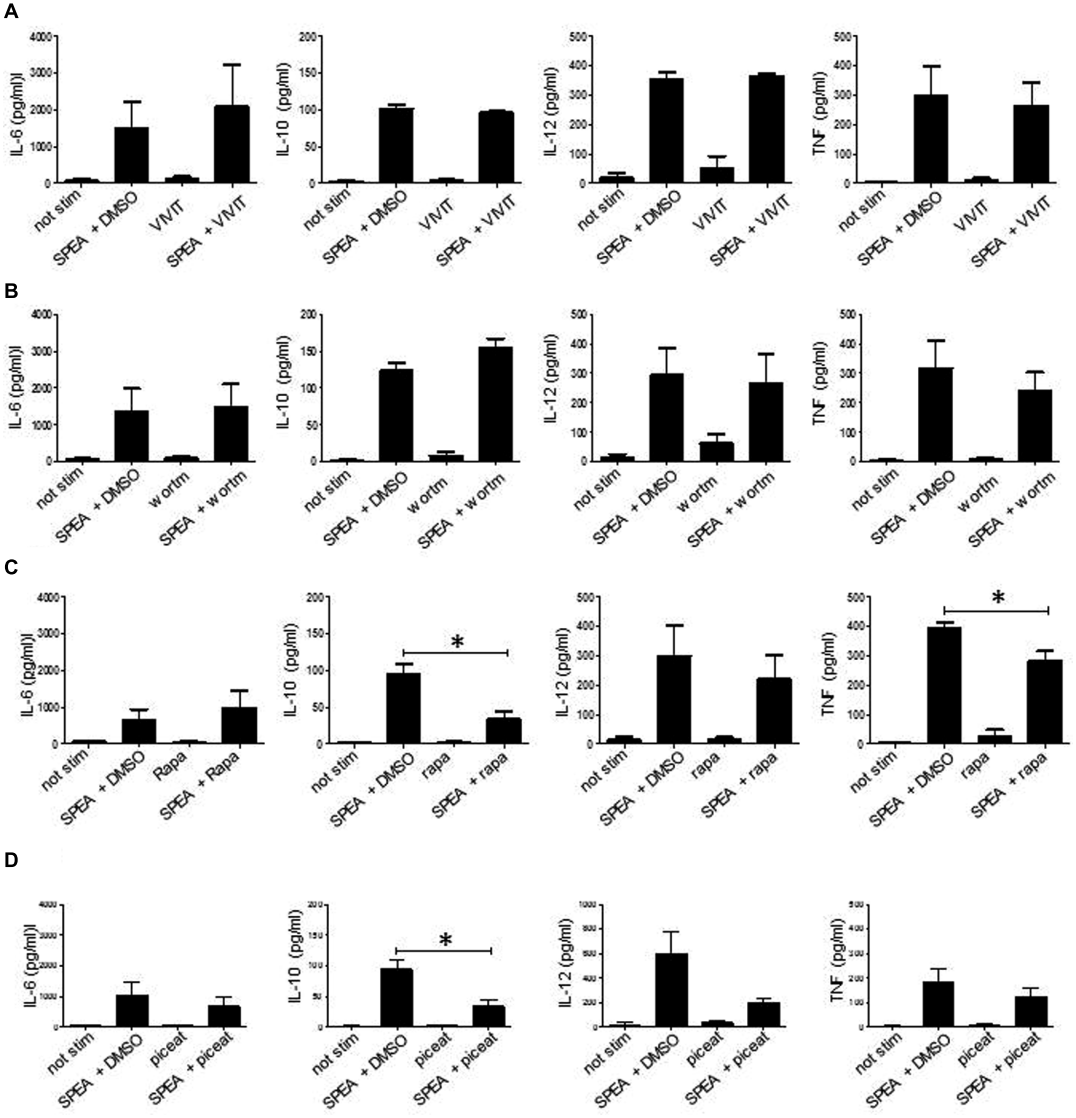
**Signaling pathways involved in SPEA- mediated activation of APC.** CD14+ monocytes were stimulated with SPEA (100ng/ml) in the presence or absence of **(A)** VIVIT (1 M), **(B)** Wortmannin (50 nM), **(C)** rapamycin (50nM) and **(D)** piceatannol (50 μM) or DMSO (equal volume to inhibitor) as solvent control. 24 h after stimulation supernatants were harvested and ELISAs were performed for IL-6, IL-10, IL-12p40 and TNF. Shown is the mean ±SD from 3 different donors, except VIVIT (n = 2). Statistical analysis was performed using a Multiple Comparison of Means (Tukey Contrasts). Significance codes: 0 ‘^∗∗∗^’; 0.001 ‘^∗∗^’; 0.01 ‘^∗^’.

It was suggested that class II-signaling induces Ca-mobilization (Mooney et al., 1990) and subsequent activation. SAg-mediated signaling in APC could thus be mediated by non-classical signaling pathways. Besides NFAT-dependent or Syk-dependent pathways which have been shown in B-cells (Scholl et al., 1992; Palkama and Hurme, 1993; Morio et al., 1994; Haylett et al., 2009) other non-canonical pathways (mTOR, PI3K) could mediate SAg stimulation of APC. To test this, we resorted to classical inhibition assays (**Figure 4**). When we tested for inhibition of the induction of pro-inflammatory and anti-inflammatory cytokines we failed to observe an effect with the inhibitors of NFAT (VIVIT) (**Figure 4A**) and PI3K (Wortmannin) (**Figure 4B**). Inhibition of mTOR pathway by rapamycin or Syk pathway by piceatannol had no effect on the induction of pro-inflammatory cytokines (TNF, IL-6, IL-12), however, rapamycin and piceatannol prevented release of IL-10 indicating a differential regulation of pro- and anti-inflammatory cytokines (**Figure 4C,D**).

**FIGURE 5:**
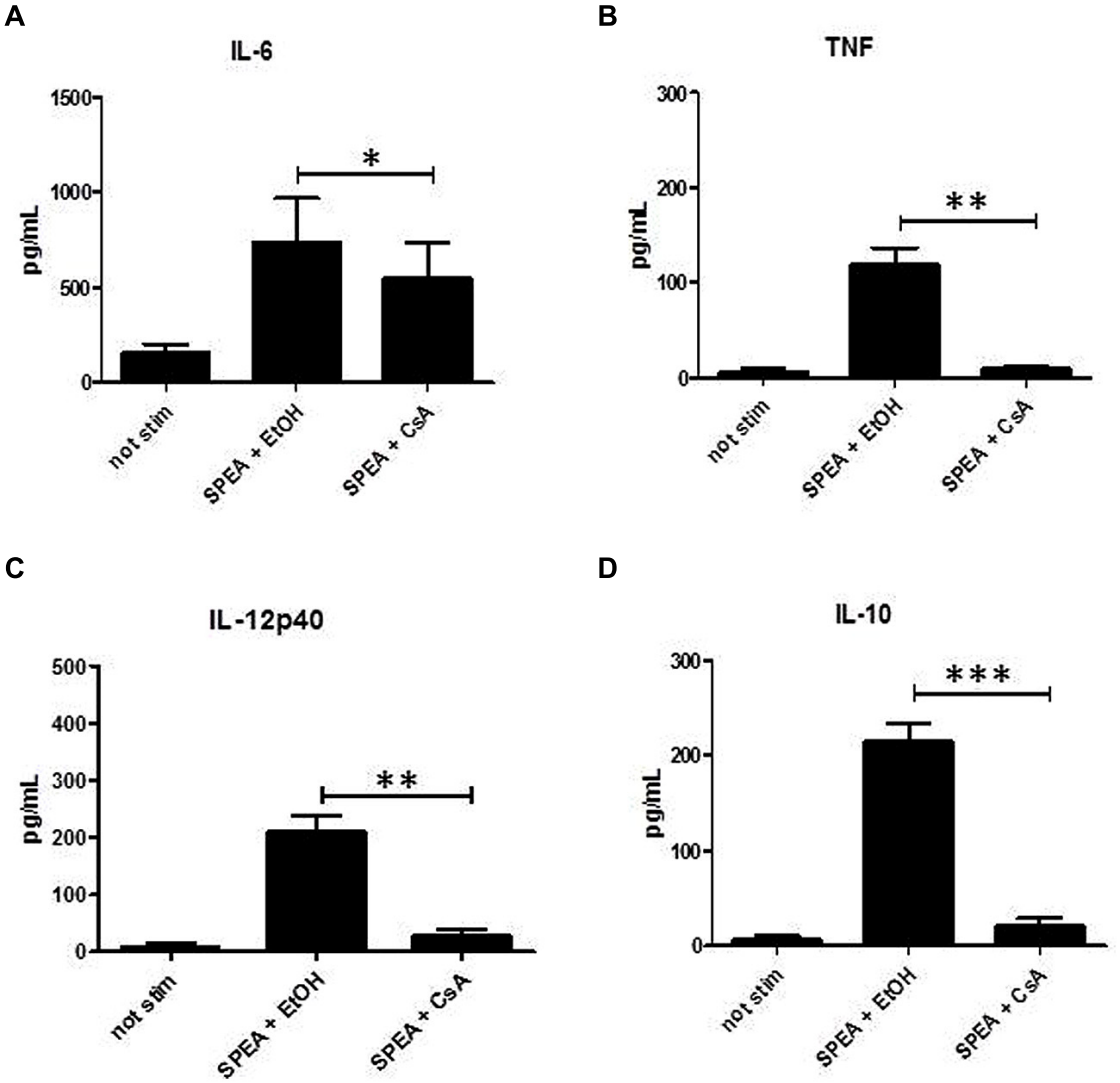
**Cyclosporine A (CsA) prevents secretion of cytokines**. CD14^+^ monocytes were stimulated with SPEA (100 ng/ml) and CsA (1 μM) or Ethanol (equal volume to CsA) for 24 h. Supernatants were harvested and analyzed by ELISA for **(A)** IL-6, **(B)** TNF, **(C)** IL-12p40 and **(D)** IL-10. Shown is the mean concentration with standard deviation of 5 different donors. Significance codes: 0 ‘^∗∗∗^’; 0.001 ‘^∗∗^’; 0.01 ‘^∗^’ (Tukey Contrasts).

**FIGURE 6:**
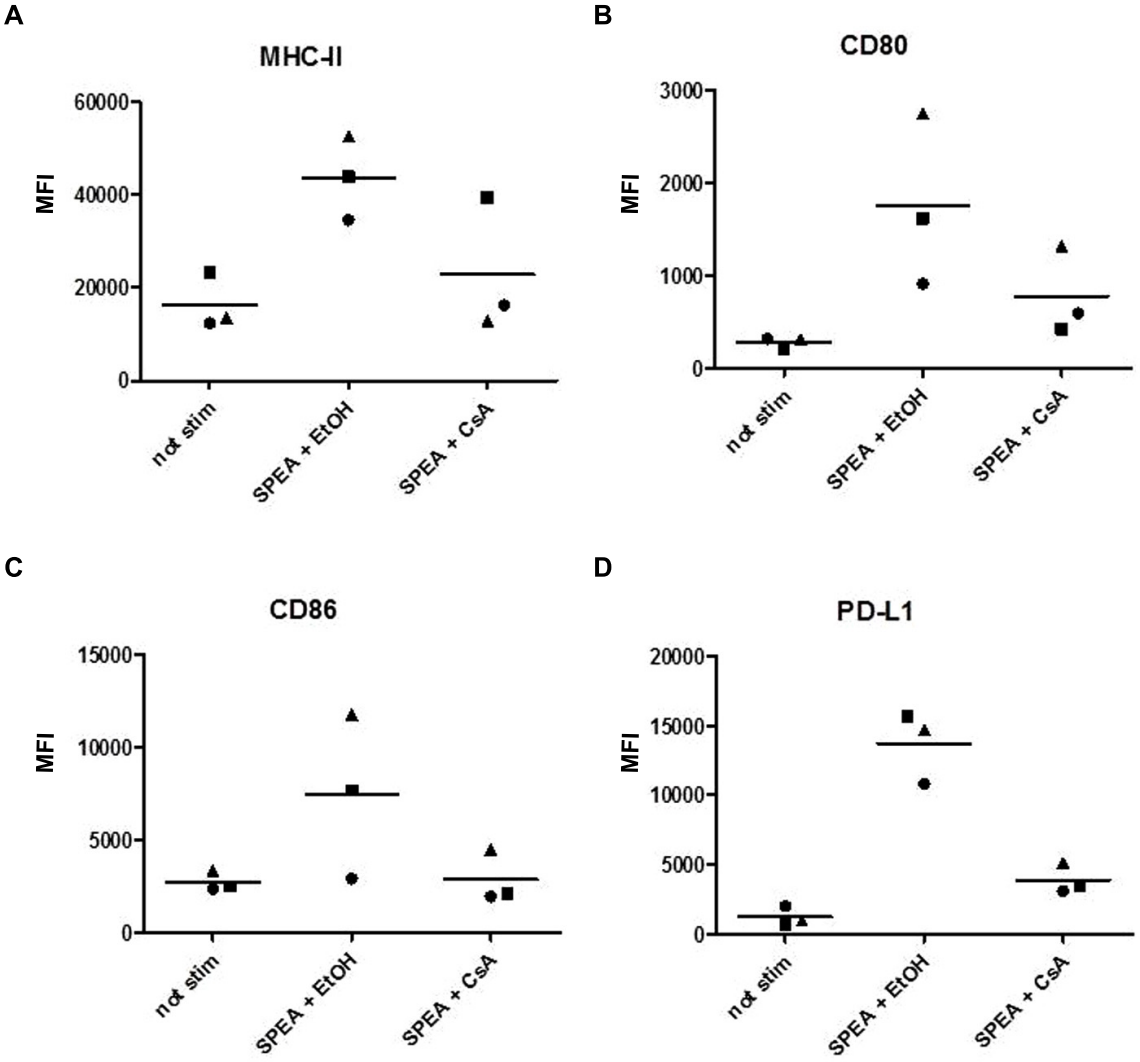
**Cyclosporine A prevents the expression of costimulatory and coinhibitory molecules**. CD14^+^ monocytes were stimulated with SPEA (100 ng/ml) and CsA (1 μM) or Ethanol (equal volume to CsA) for 24 h. Cells were harvested and analyzed for surface markers by flow cytometry **(A)** MHC-II, **(B)** CD80, **(C)** CD86 and **(D)** PD-L1. The mean fluorescence intensity of three different donors is shown.

**FIGURE 7:**
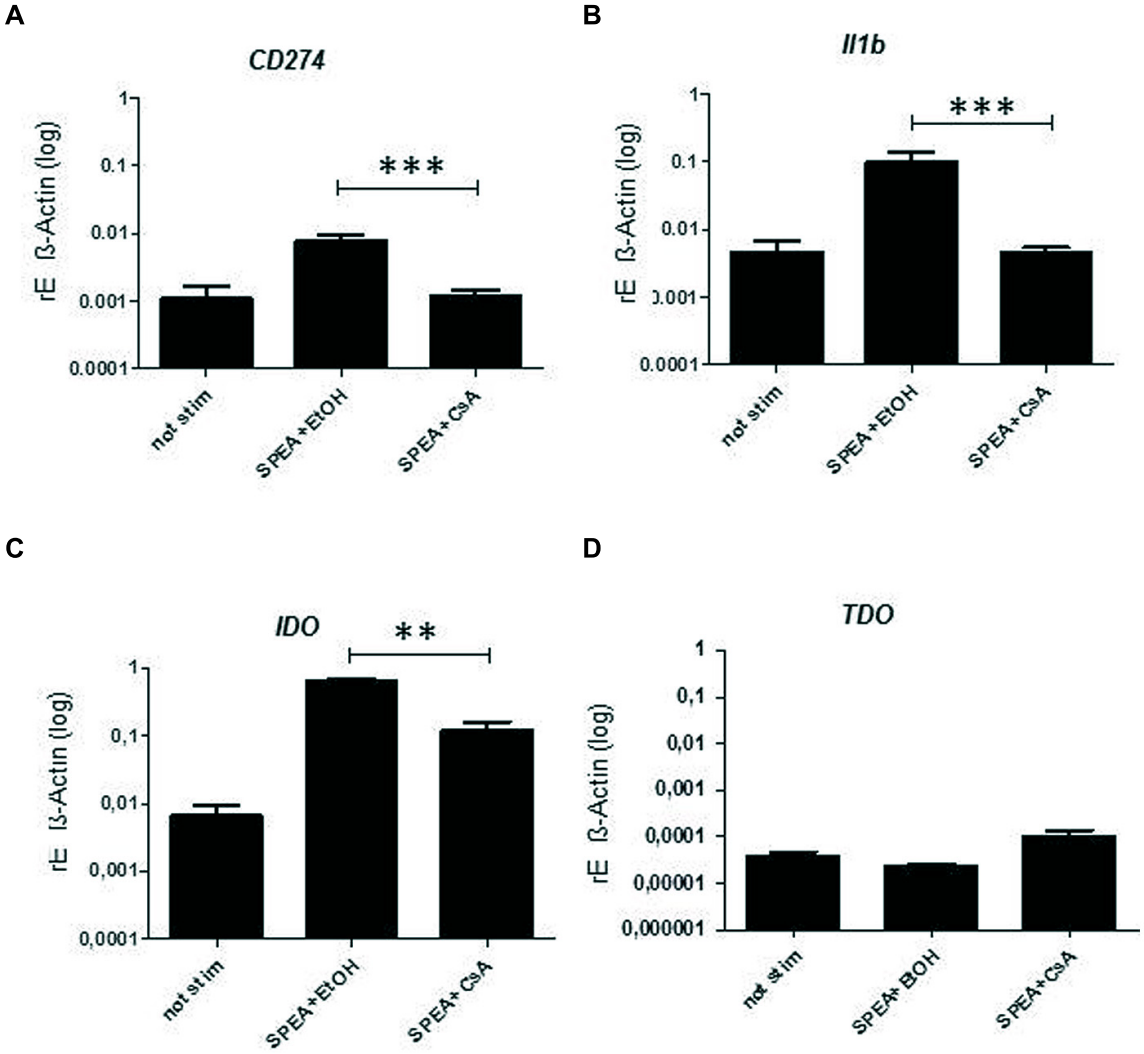
**Cyclosporine A – mediated expression of mRNA**. CD14^+^ monocytes were stimulated 24 h with SPEA (100 ng/ml) and CsA (1 μM) or Ethanol (equal volume to CsA). Quantitative real-time PCR of cDNA was performed with sequence-specific primers for **(A)** CD274, **(B)** Il1b, **(C)** IDO, **(D)** TDO. Data were normalized to ß-Actin. Shown is the mean and standard deviation of 5 donors. Statistical analysis was performed using a Multiple Comparison of Means (Tukey Contrasts). Significance codes: 0 ‘^∗∗∗^’; 0.001 ‘^∗∗^’; 0.01 ‘^∗^’.

Cyclosporine A interacts with cyclophilin and suppresses activation of T-cells and B-cells (Fischer et al., 1989). We thus hypothesized that CsA could interfere with SAg-mediated stimulation of APC. Indeed stimulation of monocytes with SPEA in the presence of CsA prevented the secretion of pro-inflammatory and anti-inflammatory cytokines significantly (**Figure 5**). Further on CsA modulated the SPEA-induced expression of co-stimulatory and co-inhibitory molecules, although not in a statistically significant mode (**Figure 6**). A similar pattern was observed for the induction of mRNA: CsA prevented the upregulation of mRNA coding for PD-L1 (**Figure 7A**), IL-1beta (**Figure 7B**), and partially reduced induction of IDO (**Figure 7C**). Accordingly, expression of TDO was not downregulated (**Figure 7D**).

**FIGURE 8:**
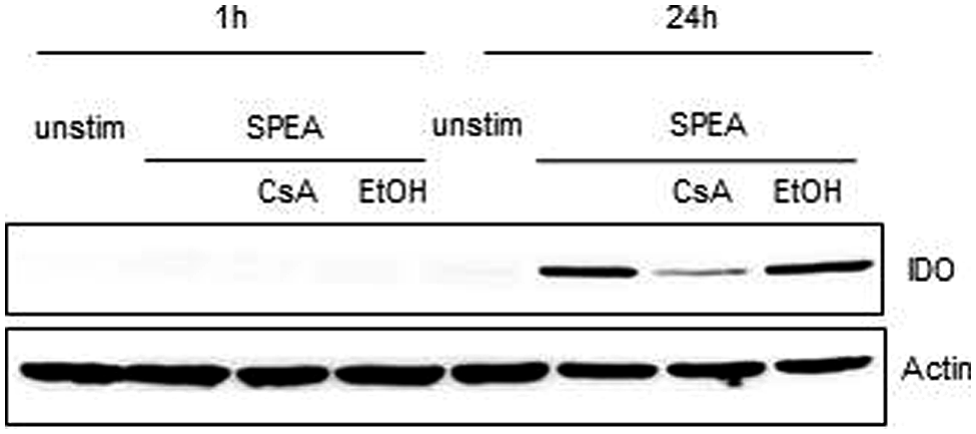
**Indoleamine 2,3-dioxygenase expression after SPEA stimulation**. CD14^+^ monocytes were stimulated for 1 h or 24 h with 100 ng/ml SPEA and 1 μM CsA (Ethanol was used equal to the amount of CsA). Cells were lysed and analyzed by western blot for IDO. ß-Actin as loading control was displayed. Shown is one representative experiment out of three.

Indoleamine 2,3-dioxygenase is a rate-limiting enzyme of tryptophan catabolism resulting in kynurenine production. Depletion of tryptophan causes halted growth of T-cells while kynurenine activates the aryl hydrocarbon (AH) receptor system leading to induction of Tregs (Opitz et al., 2011). We therefore tested for the expression of IDO and the subsequent depletion of tryptophan and production of kynurenine after SAg stimulation (**Figure 8**). In SPEA-stimulated APC IDO protein expression was detected (**Figure 8**) and the levels of kynurenine were enhanced (data not shown). Both IDO expression and kynurenine production was sensitive to inhibition by CsA corroborating the effects seen with mRNA induction (**Figure 7C**).

### STAT Activation is a Hallmark of SAg-Mediated Stimulation of APCs

**FIGURE 9:**
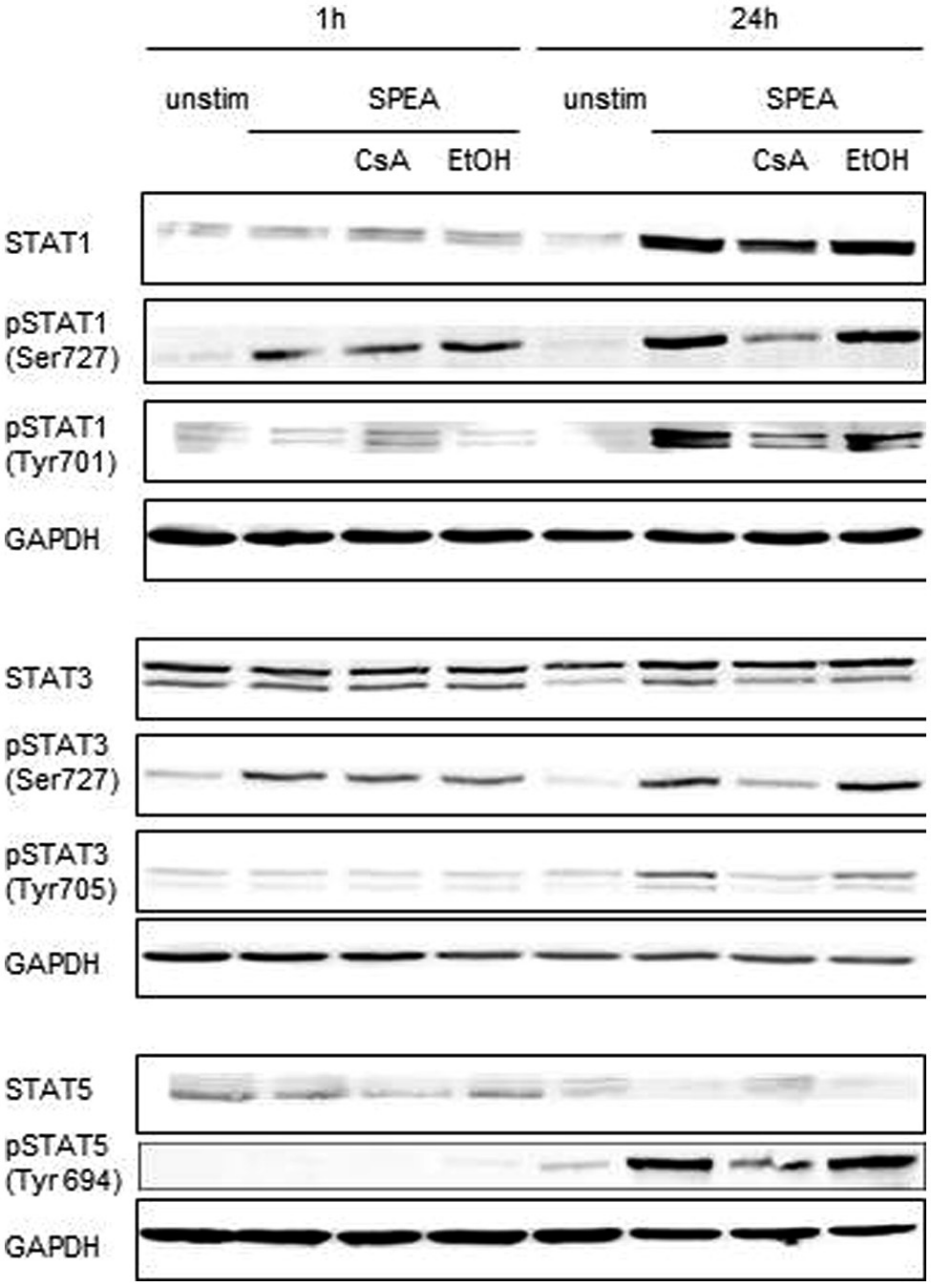
**STAT activation after SPEA stimulation**. (A) CD14^+^ monocytes were stimulated for 1 or 24 h with 100 ng/ml SPEA and 1 μM CsA (Ethanol was used equal to the amount of CsA). Cells were lysed and analyzed by western blotting with specific antibodies against STAT1, phospho-STAT1 (Ser727, Tyr701), STAT3, phospho-STAT3 (Ser727, Tyr 705), STAT5 and phospho-STAT5 (Tyr 694). GAPDH as loading control was displayed. Shown is one representative experiment out of three.

To further elucidate the signaling pathways in SAg-mediated stimulation of APC we analyzed the phosphorylation pattern of STAT molecules and MAPK after stimulation. While phosphorylation of MAPK (p38, JNK, P44/42) was not changed (Supplementary Figure S4), STAT1 and STAT3 were immediately phosphorylated, independent on CsA (**Figure 9**). STAT5 showed no phosphorylation. In contrast, after 24 h STAT1, STAT3, and STAT5 were strongly phosphorylated (**Figure 9**). The phosphorylation observed was sensitive to inhibition by CsA.

## Discussion

Superantigens are bacterial exotoxins that interact with immune cells. It was recognized long ago that activation as well as inactivation/tolerance of T-cells represent an obvious paradox. However, both reaction profiles might contribute to the immune evasion strategy of the pathogens (MacDonald et al., 1993). It is unquestionable that acute release of pro-inflammatory cytokines leads to a dysregulation of the immune and other systemic responses eventually causing septic shock. Staphylococcal or streptococcal TSS are examples of this pathogenesis (Miethke et al., 1993). They resemble cytokine storm events comparable to those observed in humans after accidentally triggering T-cells with monoclonal antibodies (Suntharalingam et al., 2006). The induction of T-cells requires binding of SAg to MHC class II molecules of APC and the followed crosslinking of T-cells to APCs (Rink et al., 1997). Binding of SAg activates APC, resulting in production of co-stimulatory cytokines and co-stimulatory molecules (Ohnishi et al., 1995).

Concomitantly inhibitory circuits are induced that subsequently dominate. After initial deletion of SAg-reactive T-cells the remaining T-cells become unresponsive and display an anergic phenotype (Kisielow et al., 1991; Wahl et al., 1993). Moreover it was shown that anergy and unresponsiveness are accompanied by the induction of CD4^+^CD25^+^ T regulatory cells (Wang et al., 1998; Noël et al., 2001; Papiernik, 2001). It is quite obvious that cytokine storm, anergy and induction of Treg induce a milieu of dysregulation and suppression that precludes a coordinated immune response and thus allows the pathogen to subvert anti-infective strategies of the host.

We show here that APC function after SAg-binding not only includes the induction of pro-inflammatory responses in terms of cytokine release and costimulation but also the induction of co-inhibitory circuits including anti-inflammatory cytokines (IL-10), co-inhibitory molecules (PD-L1) and also an induction of inhibitory effector programs (IDO). While IDO might result in an unspecific immunosupression by depleting tryptophan (Taylor and Feng, 1991) PD-L1 and IL-10 suggest an at least bystanding activity to induce Tregs (Unger et al., 2009; Wölfle et al., 2011). Moreover kynurenine an intermediate of the tryptophan metabolism produced by IDO, can further screw T-cell differentiation in direction of Tregs (Quintana et al., 2008; Gandhi et al., 2010; Opitz et al., 2011). Altogether an immunosuppressive milieu is induced which clearly foster the generation of Tregs and thus prevents an active immune response. Indeed our experiments with SPEA-treated monocytes confirm their inhibitory influence on CD3-mediated T-cell proliferation (Supplementary Figure S5). Furthermore SPEA-treated co-culture experiments with APCs and T-cells reveal a CD4^+^CD25^+^Foxp3^+^ Treg population (Supplementary Figure S6) that is functionally active and inhibits T-cell proliferation (Supplementary Figure S7).

Interestingly, negative feedback regulators for IFN signaling (SOCS-1) were also induced by SPEA (Song and Shuai, 1998; Dalpke et al., 2008; Masters et al., 2010). This could explain why APCs are refractory to stimulation with proinflammatory cytokines like IFN-gamma and thus maintain their inhibitory phenotype. We also observed a long lasting phosphorylation of STAT1 as well as STAT3 after SAg treatment of APC. Phosphorylated STAT3 induce expression of PD-L1 (Wölfle et al., 2011) and IDO (Litzenburger et al., 2014) and thus contribute to the immunosuppressive milieu. Constitutive STAT1 phosphorylation seems to contradict the immunosuppressive phenotype. Although we have not addressed this here in detail, it was shown that STAT1 action itself is under control of SOCS-1, which could explain why STAT1 phosphorylation does not necessarily induce proinflammatory cascades (Hildebrand et al., 2010).

A critical role during these processes of tolerance induction plays the APC. APC represent the scaffold to present SAg to T-cells (Dellabona et al., 1990), yet also provide other signals to the T-cells (Mehindate et al., 1995). Therefore class II-binding of SAg has to deliver a signal to the APC. Class II signaling has been described for B-cells (Mooney et al., 1990; Nabavi et al., 1992) and APCs (Chatila and Geha, 1993). Thereby different signal intermediates such as Ca^2+^ (Damaj et al., 1992), PKC (Palkama and Hurme, 1993), NFAT and MAPK (Haylett et al., 2009), and tyrosine kinases (Palkama and Hurme, 1993; Morio et al., 1994; Kanner et al., 1995) were shown. When we analyzed SAg-induced signaling using various inhibitors, we found no indication for an involvement of PI3K (Wortmannin), mTOR (rapamycin) or the tyrosine kinase Syk (piceatannol) (**Figure 4**). Since Ca^2+^ mobilization can be a consequence of SAg-activation (Damaj et al., 1992) we presumed that NFAT would be involved, yet this was not the case. In contrast all responses induced by SAg could be blocked by CsA. This was not an entirely surprising finding since we have reported recently that in an analogous stimulation model in B-cells signaling was also sensitive to inhibition with CsA but not dependent on NFAT (Ziegler et al., 2014). The sensitivity to CsA of the induction of an immunosuppressive milieu of APC fits well in older reports on the unfolding of tolerance and unresponsiveness. Sakaguchi reported that neonatal administration of CsA induced autoimmune disease in a model which was depended on Tregs (Sakaguchi and Sakaguchi, 1989). It was also shown that CsA inhibits Treg generation (Wang et al., 2006) and that immunosuppressive therapy with CsA might reduce the number of Treg after allogeneic renal transplantation (Korczak-Kowalska et al., 2007).

Cyclosporine A might interfere with the induction of Treg in two ways. Once it inhibits the Treg inducing milieu after class II binding of SAgs as described above. Secondly, CsA might affect the self-stabilizing feedback loop of Treg-APC interaction. Tregs express LAG-3 which interacts with class II molecules (Liang et al., 2008). Interaction could lead to APC activation and subsequent production of inhibitory cytokines and molecules as shown above. That would lead to a self-propagation of Tregs and thus stabilize and expand the Treg pool. Accordingly CsA would prevent this loop by inhibiting the response to class II crosslinking. It has been shown that blockade of PD-L1 and LAG-3 rapidly cleared infection with plasmodia (Butler et al., 2012), indicating that during infection this loop is operative and could be manipulated.

Taken together we show here that APC triggered by SAg are not only responsible for the initial induction of pro-inflammatory responses but are also crucial for the induction and maintenance of unresponsiveness and anergy. Paradoxically the T-cell immunosuppressive drug CsA prevents in SAg triggered APC the manifestation of the immunosuppressive program, indicating that CsA could be utilized to prevent SAg-induced anergy and unresponsiveness.

## Author Contributions

AS, SF, KH, and DH designed the study. DH and KH wrote the final manuscript. AS, SF performed the experiments. All authors read the manuscript and discussed the results.

## Conflict of Interest Statement

The authors declare that the research was conducted in the absence of any commercial or financial relationships that could be construed as a potential conflict of interest.
