# Supplementary material for: T-cell activation or tolerization: the Yin and Yang of bacterial superantigens
Source: Front Microbiol. 2015 Oct 20;6:1153. doi: 10.3389/fmicb.2015.01153 (PMC4611159; doi:10.3389/fmicb.2015.01153)
Supplement: Supplementary file 1 [file Presentation_1.PDF]

Supplementary Material

T-cell activation or tolerization: the Yin and Yang of bacterial superantigens

Aline Sähr [1,2], Sandra Förmer[1,2], Dagmar Hildebrand[1], Klaus Heeg[1]*

[1]Medical Microbiology and Hygiene, Department of Infectious Diseases, University Hospital Heidelberg, Heidelberg, Germany

[2] both contributed equally

*** Correspondence:** Klaus Heeg, MD, Medical Microbiology and Hygiene, Department of Infectious Diseases, University Hospital Heidelberg, Im Neuenheimer Feld 324, Heidelberg 69120, Germany

klaus.heeg@med.uni-heidelberg.de

Supplementary Methods

IL-1ß ELISA

Cell-free supernatants were harvested 24hours after stimulation and analyzed for IL–1ß by ELISA kits from Becton Dickinson (OptEIA; Becton Dickinson, Heidelberg, Germany) according to the manufacturer's instructions.

T-cell isolation

Human peripheral blood mononuclear cells were isolated from fresh blood or buffy coats by density gradient centrifugation (Pancoll 1.077 g/mL; PAN Biotech). Untouched CD4-positive T-cells were selected by magnetic-associated cell sorting (CD4+ T Cell Isolation Kit, Miltenyi Biotec) and AutoMACS technology.

Co-culture experiments

$CD14^+$ monocytes were pulsed with anti-CD3 antibody (500ng/ml) and stimulated with SPEA (100ng/ml). After 24 hours, cells were washed with PBS and cultured together with T cells ($1x10^6$/ml monocytes, $2x10^6$/ml T cells) in fresh medium. 48 hours later the proliferation of cells was analyzed by flow cytometry.
$CD4^+$ T cells were co-cultured for 6 days with APCs in the presence of SPEA (10ng/ml). After 6 days Tregs were harvested (APCs stick to the bottom) and add to a fresh co-culture of CFSE-stained $CD4^+$ T cells and unstained APCs from the same donor, stimulated with SPEA (10ng/ml). Tregs were added in a ratio from 1:1 to 1:10. Proliferation (decreasing FITC signal) was analyzed 4 days after stimulation by flow cytometry. As controls unstimulated and SPEA-treated co-cultures (CFSE-labeled T cells/ unstained APCs) were analyzed.

CFSE staining

T-cells were stained with carboxyfluorescein succinimidyl ester (2.5 million cells/ml, 5 µM CFSE, Biolegend) for 10 min at room temperature in the dark, before the reaction was stopped with cold medium containing 10% FCS.

Foxp3 staining

$CD14^+$ monocytes were stimulated with SPEA (10ng/ml) and co-cultured with $CD4^+$ T cells 24h after treatment ($5x10^5$ monocytes, $5x10^5$ T cells). On day 6 cells were surface stained for CD4 (BD, clone RPA-T4), CD25 (biolegend, clone M-A251) before intracellular staining for Foxp3 using the Foxp3/Transcription Factor Staining Buffer Set (eBioscience). Cells were fixed and permeabilized for 45 minutes at 4°C with Fixation/Permeabilization buffer. After 2 washing steps using Permeabilization buffer, unspecific binding was blocked with FcR block (Miltenyi Biotec). The intracellular staining followed using Foxp3 antibody (eBioscience, clone PCH-101) for 30 minutes at 4°C. After 3 washing steps cells were analyzed by flow cytometry.

Supplementary Figures

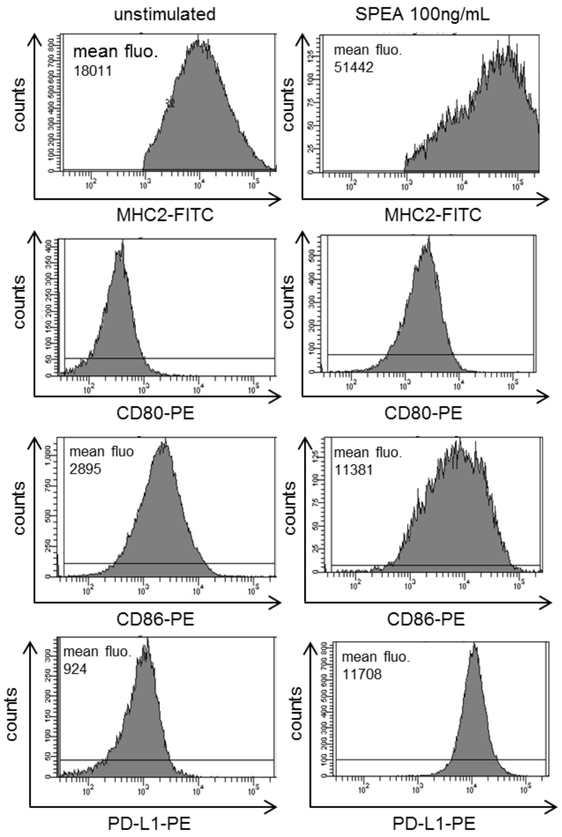

Supplementary Figure 1 SPEA-induced surface molecules.
CD14^{+} monocytes were stimulated with 100ng/ml SPEA for 24hours. Cells were harvested and analyzed for surface markers by facs. Shown are the histograms of CD14-gated cells. Mean fluo. = mean fluorescence of the gated cells. Experiment was repeated three times with comparable results.

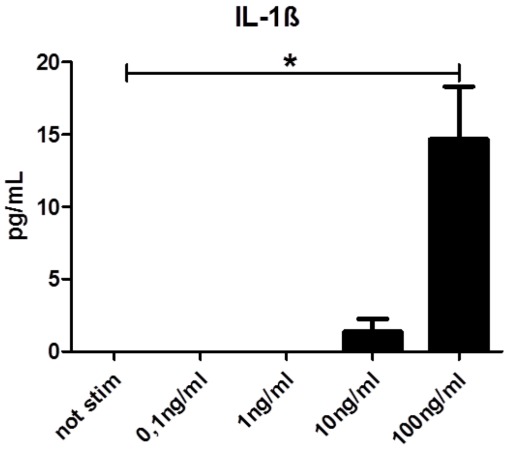

Supplementary Figure 2 SPEA-induced release of activated IL-1β.
CD14^{+} monocytes were stimulated 24hours with increasing concentrations of SPEA. Cell-free supernatants were harvested 24hours after stimulation and analyzed for IL–1ß by ELISA. Shown is the mean and standard deviasion of four donors. Statistical analysis was performed using a paired student's t-test (* $p \leq 0.05$).

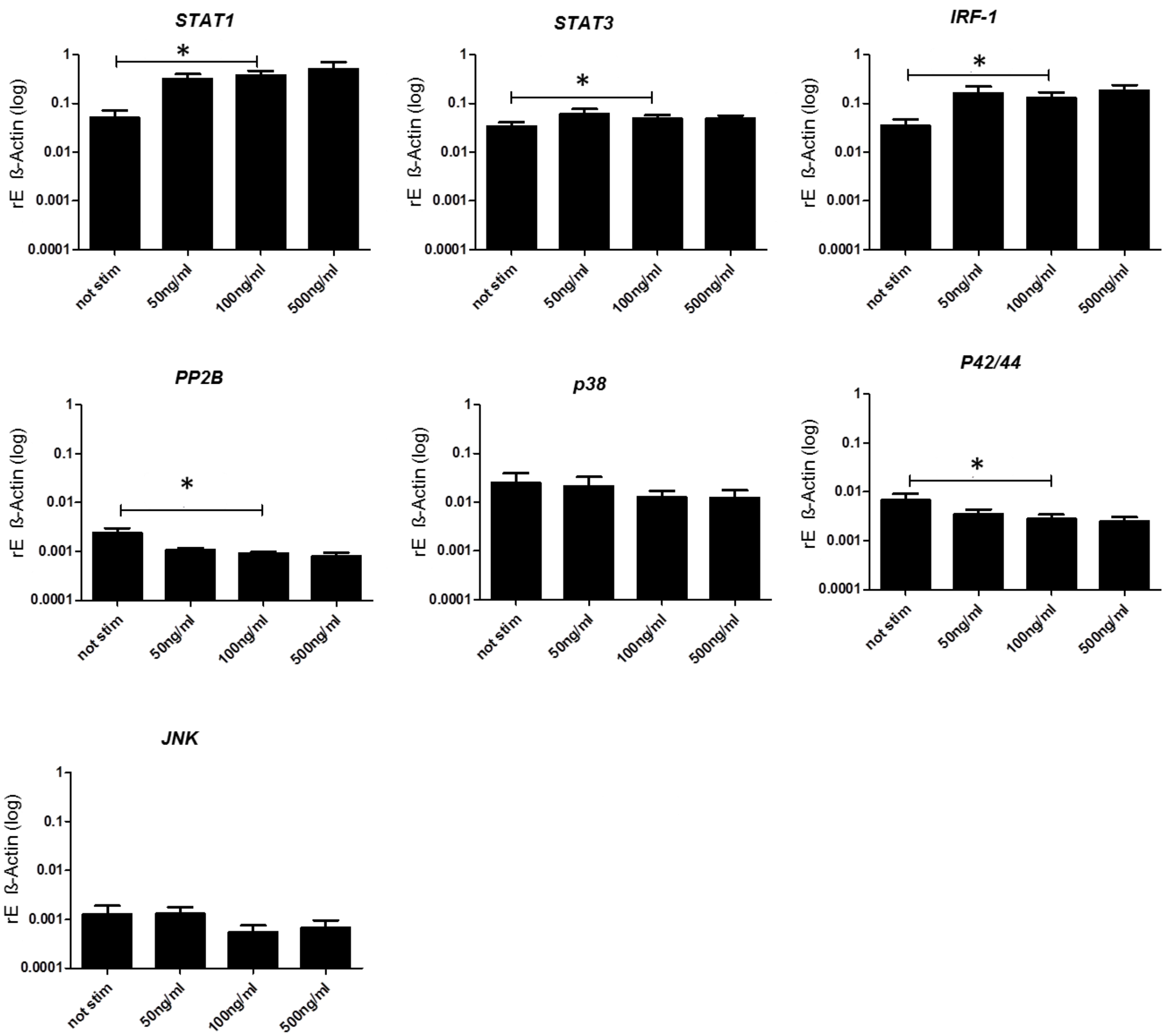

Supplementary Figure 3 SPEA-induced mRNA expression of inhibitory pathways.
$CD14^+$ monocytes were stimulated 24hours with increasing concentrations of SPEA. Quantitative real-time PCR was performed for *STAT1*, *STAT3*, *IRF-1*, *PP2B* (Calcineurin A), and the MAP-kinases *p38*, *p44/42* and *JNK*. Shown is the mean of induction compared to *ß-Actin* (SD, n=3). Statistical analysis was performed using a paired student's t-test (* $p \leq 0.05$).

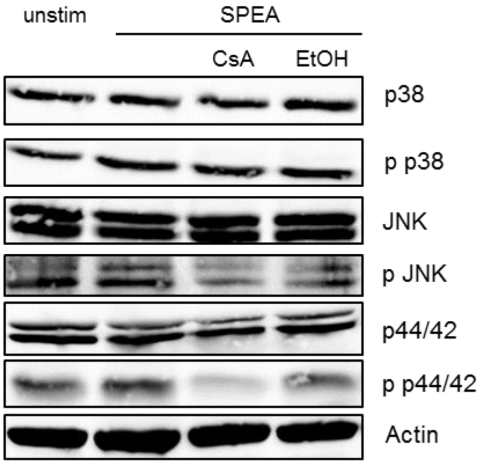

Supplementary Figure 4 MAPK activation after SPEA stimulation.
CD14$^+$ monocytes were stimulated for 24hours with 100ng/ml SPEA and 1µM Cyclosporine A (Ethanol was used equal to the amount of CsA). Cells were lysed and analyzed by western blotting with specific antibodies against p38, JNK and p44/42. Shown is one representative experiment out of three.

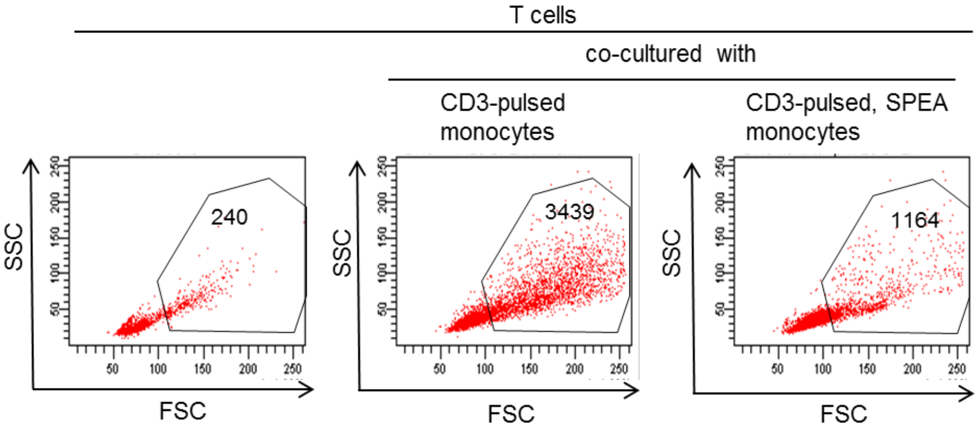

Supplementary Figure 5 SPEA- treated monocytes inhibit CD3-mediated T-cell proliferation. $CD14^+$ monocytes were pulsed with anti-CD3 antibody (500ng/ml) and stimulated with SPEA (100ng/ml). After 24 hours and change of media untouched $CD4^+$ T-cells were added. 48 hours later cells were analyzed for proliferation by flow cytometry. Shown are gated CD4-positive T-cells. The numbers display the events (cell number) in the gate. X-axis: FSC (forward scatter), y-axis: SSC (side scatter). Shown is one representative experiment out of three.

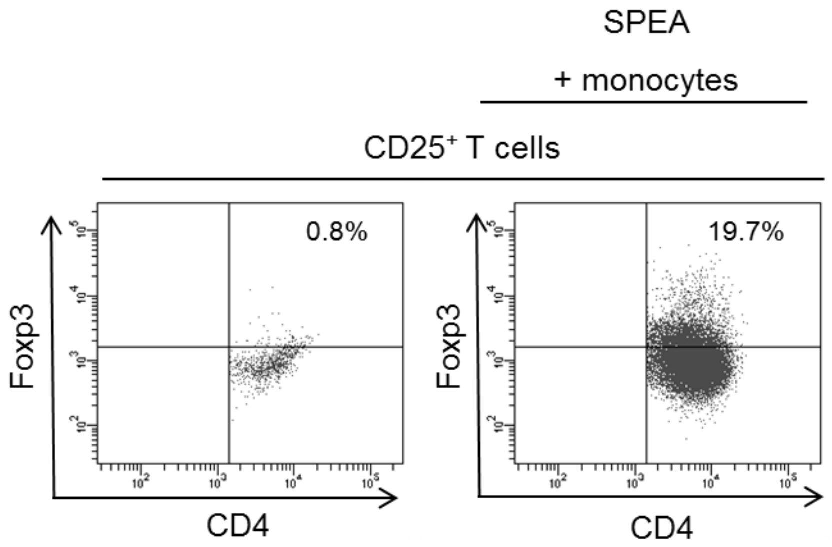

Supplementary Figure 6 SPEA treatment induces Foxp3$^+$ cells in T-cell/monocyte co-culture. CD14$^+$ monocytes were stimulated with SPEA (10ng/ml). After 24h untouched CD4$^+$ T cells were added. Cells were harvested after six days and analyzed for surface markers CD4, CD25 and intracellular for Foxp3 by flow cytometry. Gated CD4$^+$ CD25$^+$ T-cells and their percentage of Foxp3 expression are shown. The experiment was repeated with comparable results.

A

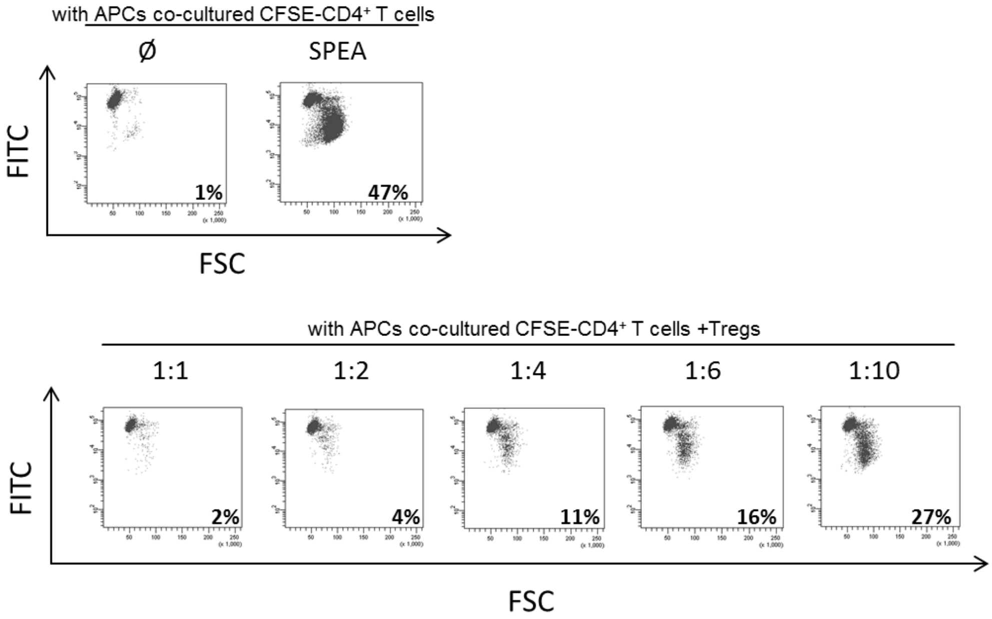

B

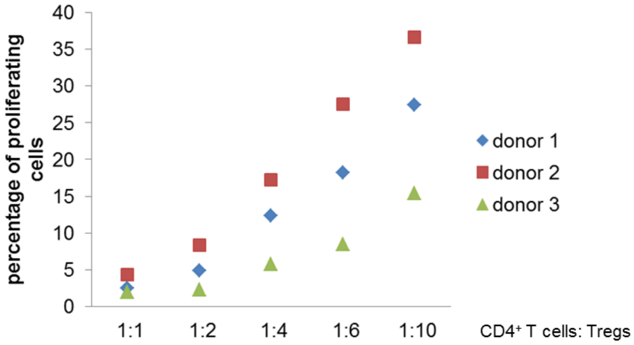

Supplementary Figure 7 SPEA-induced Tregs inhibit T-cell-proliferation

CD4$^+$ T-cells were co-cultured for 6 days with APCs in the presence of SPEA (10ng/ml). After 6 days Tregs were harvested and add to a fresh co-culture of CFSE-stained CD4$^+$ T-cells and unstained APCs from the same donor, stimulated with SPEA (10ng/ml). Tregs were added in the indicated ratios. A) Proliferation was analyzed 4 days after stimulation by flow cytometry. As controls unstimulated and SPEA-treated co-cultures (CFSE-labeled T-cells/ unstained APCs) without Tregs were analyzed. The percentages indicate the number of T-cells that proliferated. Shown is one representative experiment out of three and the percentage of proliferating cells. B) The graph shows the quantification (percentage) of proliferating CFSE-T-cells of three donors.
